# Getting to the Heart of the Matter: Exploring the Intersection of Cardiovascular Disease, Sex and Race and How Exercise, and Gut Microbiota Influence these Relationships

**DOI:** 10.31083/RCM26430

**Published:** 2025-02-20

**Authors:** John J. Guers, Kevin S. Heffernan, Sara C. Campbell

**Affiliations:** ^1^Department of Health Sciences and Nursing, Rider University, Lawrenceville, NJ 08648, USA; ^2^Department of Biobehavioral Sciences, Teachers College, Columbia University, New York, NY 10027, USA; ^3^Department of Kinesiology and Health, The State University of New Jersey, New Brunswick, NJ 08901, USA; ^4^Centers for Human Nutrition, Exercise, and Metabolism, Nutrition, Microbiome, and Health, and Lipid Research, Rutgers, The State University of New Jersey, New Brunswick, NJ 08901, USA

**Keywords:** cardiovascular disease, ethnicity, microbiome, heart, intestine, physical activity

## Abstract

Cardiovascular disease (CVD) is the leading cause of death worldwide, with physical inactivity being a known contributor to the global rates of CVD incidence. CVD incidence, however, is not uniform with recognized sex differences as well and racial and ethnic differences. Furthermore, gut microbiota have been associated with CVD, sex, and race/ethnicity. Researchers have begun to examine the interplay of these complicated yet interrelated topics. This review will present evidence that CVD (risk and development), and gut microbiota are distinct between the sexes and racial/ethnic groups, which appear to be influenced by acculturation, discrimination, stress, and lifestyle factors like exercise. Furthermore, this review will address the beneficial impacts of exercise on the cardiovascular system and will provide recommendations for future research in the field.

## 1. Introduction and a Brief Overview of Topics 

Cardiovascular disease (CVD) remains the leading cause of mortality and morbidity worldwide [1]. CVDs include atherosclerosis, myocardial infarction, stroke, heart failure, and hypertension, among others. Risk factors for CVD can be categorized as either modifiable (habitual alcohol and tobacco use, high blood lipids, high blood pressure, excess adiposity/body fat, poor glucose control/diabetes, physical inactivity, and high-fat “Western” diet) or nonmodifiable (age, biological sex, genetics). Physical inactivity is a known contributor to the global rates of CVD [2]. The United States “Physical Activity Guidelines for Americans” recommend that adults engage in 150–300 minutes of moderate or 75 minutes of vigorous physical activity each week [2].

This narrative review will build on our previous work by presenting novel data that shows a clear relationship between CVD, exercise, sex, race and gut microbiota. Specifically, we highlight how biological sex and race impact gut microbiota and how exercise can be used to improve gut health while minimizing disparities. These factors are all linked in a complicated system that ultimately can strongly influence cardiovascular health. This review will provide a brief outline of each topic, take a deep dive into the impacts of exercise on CVD with considerations for sex, race and gut microbiota, truly getting to the heart of the matter.

## 2. Topical Overviews

### 2.1 Exercise Promotes Longevity and Health

It is well known that exercise preserves health. Studies conducted as early as the 1910’s highlight the protective effects of manual labor on degenerative diseases [3]. Similar reports reinforced the notion that physical activity can help prevent disease [4]. More recently, studies have shown that aerobic capacity correlates with an increased lifespan and increased “healthspan” [5]. Exercise is known to decrease all-cause mortality, and we know that cardiorespiratory fitness correlates with longevity [6]. Over the past several decades researchers have become interested in which potential mechanisms are responsible for these protective effects. For the purposes of this paper, we will focus on the mechanisms involved with exercise-induced protection of the cardiovascular system.

### 2.2 Gut Microbiota and Exercise

The gut microbiota consists of trillions of microbial cells such as bacteria, fungi, viruses, and archaea [7]. Regarding gut bacteria, there are over 1100 genera, and approximately 90% fall under the phylum Bacteroidota and Bacillota (formerly known as Bacteroidetes and Firmicutes [8], respectively) while, the minority of gut bacteria are Pseudomonadota, Actinomycetota, Fusobacteriota, and Verrucomicrobiota (formerly known as Proteobacteria, Actinobacteria, Fusobacteria, Verrucomicrobia [8], respectively) phyla [9]. Commonly observed in a healthy gut microbiota is a decreased Bacillota to Bacteroidota ratio, stable community, and greater species diversity [10].

The gut microbiota is now recognized as being critical for the maintenance of optimal human health. When the gut microbiota is in symbiosis with the host, microbes can promote health. However, when in dysbiosis (unbalanced gut microbes) with the host, the bacteria can contribute to chronic disease. In a healthy host, the gut microbiota favorably affects digestion, nutrient absorption, and production of folate, vitamins, and short chain fatty acids (SCFAs).

Our lab [10], and others [11, 12, 13] have examined the link between the gut microbiota and exercise in animal models. The gut microbiota of sedentary individuals differs from active individuals [14, 15, 16, 17]. Results from humans and animal studies clearly show that exercise is central to healthful aging, improves the diversity of microbes within the Bacillota phylum [10, 13, 14], and increases the abundance of beneficial bacteria such as *Roseburia intestinalis*, *Faecalibacterium prausnitzii*, and *Akkermansia muciniphila* [15, 18].

In addition, the gut microbiota appears to adapt to the unique demands of exercise [19, 20, 21]. Changes in the gut microbiota that occur with exercise generate metabolites that further provide the host with performance advantages [19, 20, 21, 22, 23, 24]. Athletes typically have improved carbohydrate metabolism, higher tolerance to oxidative stress, greater insulin sensitivity, enhanced muscle tissue repair, and greater energy harvesting [14, 25, 26, 27].

Moreover, results from antibiotic and germ-free mouse models demonstrate a bidirectional relationship between gut microbiota and exercise. Results show that gut microbiota must be intact for exercise performance and various aspects of maintenance of exercise training but perhaps not for adapting to exercise training [12, 19, 20, 21, 22, 23, 24, 28].

In summary, habitually exercise-trained individuals have a beneficial gut microbiota. Additionally, sedentary individuals who undertake exercise training can improve the abundance of beneficial gut microbes. Importantly, exercise-induced microbial changes in human studies are observed across the lifespan and are seen in both men and women. It is important to underscore that the favorable gut modifications that come with habitual exercise training are lost with cessation of exercise (“use it or lose it”). In conclusion, an intact gut microbiota must be present to fully adapt to exercise-induced training adaptations, including muscle hypertrophy.

### 2.3 Sex and Exercise Differences

There are established sex differences in heart size, stroke volume, and hemoglobin content contributing to exercise performance [29, 30, 31]. Among humans, sex differences in heart size do not manifest until puberty. By adulthood, hearts are approximately 30% larger in males compared to females, primarily due to greater myocyte hypertrophy among males [32]. These observed sex-based differences in heart size are the primary factors contributing to larger stroke volume among males compared to females [33, 34, 35]. However, there does not appear to be a difference in maximum heart rate by sex [33]. Hemoglobin concentration in blood is higher for males compared to females, contributing to sex differences in oxygen carrying capacity [36]. Although males have larger muscle fibers and more capillaries per fiber, capillary density does not differ between sexes [37]. Furthermore, while skeletal muscles of men are usually stronger and more powerful than women, men are often more fatigable than women for sustained or intermittent isometric contractions performed at a similar relative intensity [38]. Importantly, these fundamental differences between biologic males and females emerge at the onset of puberty, suggesting that sex hormones may be responsible for conferring sex-based differences. This is relevant because exercise motivation, particularly in females, has been shown to be regulated by estrogen. Krause *et al*. [39] demonstrated that in estrogen deficiency there was reduced melanocortin-4 signaling which lowered the drive to exercise, illuminating the power of estrogen during the reproductive cycle in motivating behavior and maintaining an active lifestyle in women. Intriguingly, estrogen deficiency (menopause) is also when CVD risk increases [40], meaning not only are women at high risk of CVD, but they may be less likely to want to engage in exercise which would help in the prevention of CVD and other metabolic risk factors.

### 2.4 Sex Differences in Gut Microbiota Considering Lifespan

Studies comparing compositional differences in the microbiota between males and females often find differences between each sex, but not always [41]. This may indicate that the sex differences are context-dependent. For example, in several studies, compositional differences were described as females having higher levels of *Clostridium* from the Bacillota (formerly Firmicutes) phylum and males having higher levels of *Prevotella* from the Bacteroidota (formerly Bacteroidetes) phylum and *Lactobacillus* from the Bacillota phylum [42, 43, 44]. Other observations include males having less microbial diversity compared to females [42]. These compositional differences are not always consistent between the sexes, particularly when a study alters an additional factor like diet [42].

#### 2.4.1 Birth and Childhood

A variety of factors impact microbiota in the early years of life including mode of birth, breastfeeding or formula feeding, antibiotic treatment, genetics, sex, and more [41]. Consequently, these microbes likely affect human development in a sex-dependent manner. Even from birth, some studies show different microbial communities between males and females [42]. For example, females delivered by asthmatic mothers are prone to *Bacteroidaceae* microbes compared to males that tend to harbor *Lactobacilli* [45]. Another example of early sex differences observing 300 infants is the temperament of males appears to be more positive when *Bifidobacterium* of the Actinomycetota (formerly Actinobacteria) phyla and *Clostridiaceae* of the Bacillota phyla are present [46]. Female members that have gut communities with *Veillonella* tend to be more risk averse [46]. Using reverse-transcriptase qPCR a study showed that boys had higher abundance of several *Bifidobacterium* spp. over three years [47]. A study examined how normal weight pre-puberty girls have increased Bacteroidota compared to obese girls [48]. Interestingly, these differences were not seen in boys of the same age [48]. Obesity in girls of this group had more developed adrenal glands and an underexpression of gonadal estradiol, the predominant estrogen [49]. Boys in this group had increased dehydroepiandrosterone (DHEA) [49]. Given that other studies have linked estrogen levels with certain groups of microbes, it would suggest that these girls could have gut microbes that play a role in estrogen-driven diseases.

#### 2.4.2 Puberty

During puberty, the difference in levels of sex hormones between males and females increases, and the effects they have on the microbiome appear to be more prominent as well [50]. For example, in a human twin study of teenagers, there was greater dissimilarity of the gut microbiota between opposite-sex twins than same-sex twins during puberty [51]. In another study using mice, the alpha-diversity of females changed significantly compared to males after puberty and the sex-related compositional differences disappeared after these male mice were castrated [52]. Interestingly, in a study by Yuan *et al*. (2020) [53] there was no difference in alpha-and beta-diversity of girls and boys before puberty, but there was an association of certain microbes to testosterone including *Adlercreutzia*, *Ruminococcus*, *Dorea*, *Clostridium*, and *Parabacteroides*. Similarly, male mice undergoing a gonadectomy were administered testosterone and subsequently, did not exhibit the microbiota changes [52]. Another group of mice that had a gonadectomy that did not receive testosterone supplementation did exhibit microbial changes [52]. This highlights testosterone as a key factor in microbial change. Similar studies performing ovariectomies on mice showed changes in microbiota including a reduction of Pseudomonadota (formerly Proteobacteria), higher *Akkermansia*, and a decreased ratio of Bacillota to Bacteroidota [54].

#### 2.4.3 Adulthood

During adulthood, estrogen and testosterone are described as potent modifiers of the human body and the microbiota [55]. And due to the different concentrations of sex hormones in males and females, the microbiota and its effects are modulated in a sex-dependent manner [55]. The adult microbiota is also characterized as being more stable compared to other stages of life [42]. In a human study of 516 Japanese males and females, *Prevotellaceae* was more abundant in males and *Ruminococcaceae* was more abundant in females [44]. The microbiota from 91 pregnant women were transplanted via fecal microbiota transfer (FMT) into germ-free (GF) mice in the 1st and 3rd trimester [56]. Mice receiving FMT from third trimester (T3) showed pregnancy-like effects like increased adiposity and insulin sensitivity, but FMT from first trimester (T1) did not show these effects [56]. Additionally, there was no correlation between the microbiota compared to estrogen levels throughout the menstrual cycle of 17 females [57]. Importantly, adulthood is when many diseases can progress, and this can have sex-dependent effects on the microbiota as well. In a study by Mahnic *et al*. (2018) [58], they also found higher levels of *Bacteroides* and *Prevotella* in males compared to females. To understand these relationships fully, the mechanisms that influence them should be investigated.

#### 2.4.4 Old Age

As people age, the microbial changes between males and females become less prominent [42]. It is important to note that this is also when male and female hormone levels become more similar [41]. These events are likely not a coincidence. In a study by Santos-Marcos *et al*. (2018) [59], the microbiota of human males and post-menopausal females were compared to measure any differences between each sex. The Bacillota/Bacteroidota ratio was different between males and females as well as the amount of saccharolytic activity [59]. More specifically, pre-menopausal women versus post-menopausal women and pre-menopausal females versus males were most different [59]. Given that estrogen levels are greatly reduced in post-menopausal women, the data suggests that the changes in the microbiota are influenced by the changes in sex hormones [59]. Interestingly, Deltaproteobacteria in the cecum increased in abundance as mice aged [60]. This raises the question of how age may impact the microbiota differently depending on where along the gastrointestinal tract the sample is taken.

### 2.5 Race, Sex, and Exercise Differences 

According to the 2022 Centers for Disease Control, National Center of Health Statistics Data Brief on physical activity in the United States (US) the percentage of adults who met the guidelines for both aerobic and muscle-strengthening activities varied by race and Hispanic origin [61]. In general, in 2020, 24.2% of adults aged 18 and over met the 2018 Physical Activity Guidelines for Americans for both aerobic and muscle-strengthening activities [61]. When accounting for race/ethnicity Hispanic men (23.5%) were less likely to meet both physical activity guidelines than non-Hispanic White (30.5%), non-Hispanic Asian (30.2%), and non-Hispanic Black (29.7%) men [61]. Non-Hispanic White women (24.3%) were more likely to meet both guidelines than Hispanic (18.0%), non-Hispanic Asian (16.7%), and non-Hispanic Black (16.5%) women [61]. Across all race and Hispanic-origin groups, men were more likely than women to meet the guidelines for both aerobic and muscle-strengthening activities [61]. The percentage of men who met both physical activity guidelines increased as family income increased, from 16.2% of men with a family income of less than 100% of the federal poverty level (FPL), to 20.0% of men with income at 100%–199% of FPL, and 32.4% of those with income at 200% of FPL or more [61]. The percentage of women who met both physical activity guidelines increased as family income increased, from 9.9% of women with family income less than 100% of FPL, to 13.6% of women with income at 100%–199% of FPL, and 25.9% of those with income at 200% of FPL or more [61]. Across all income groups, men were more likely than women to meet the guidelines for both types of activity [61].

### 2.6 Racial Disparities and Gut Microbiota 

Currently, human gut microbiota studies have had a narrow focus or simply describe broad population-level changes to gut communities in response to environmental variation. As such, only a few studies have been designed to address gut microbiota variation in relation to structural inequities, and even fewer have attempted to link host health to socially attributed variations in the gut microbiota [62, 63, 64, 65, 66]. Nevertheless, the small but existing literature does provide accumulating evidence that the social and environmental factors that contribute to health inequities may also predict gut microbiota characteristics. For example, measures of socioeconomic status (SES) across globally diverse populations, have been associated with a distinct gut microbiota in both adults [66, 67, 68] and children [69, 70, 71, 72, 73]. Similarly, the gut microbiota consistently varies with race (e.g., Asian, Black, Hispanic, White) and/or ethnicity/ancestry (Arapaho, Cheyenne, Dutch, Ghanaian, Moroccan) in adults [62, 63, 65, 74] and children [70, 71, 75, 76].

There is strong evidence linking structural inequities to gut microbiota variation in the context of SES. For example, neighborhood SES has been shown to explain 12–25% of the variation in adult gut microbiota composition, after adjustment for demographic and lifestyle factors, and was positively correlated with gut microbiota diversity [67]. Similar results noting an association between neighborhood SES and gut microbiota diversity were also obtained utilizing a discordant-twin analysis, which minimizes the possibility of confounding by shared genetic or family influences [68]. Finally, it has been shown that the relative abundance of taxa, accounting for 38.8% of the gut microbiota, varies in relation to indices of wealth appraised as personal yearly income and spending [66].

Despite the important contributions of these findings, most gut microbiota studies in minoritized populations do not operationally define structural inequities. Furthermore, race and ethnicity/ancestry are often incorrectly conflated. Whether the gut microbiota is impacted more by the personal lived experiences of perceived racism and discrimination (internalization) versus overt structural/systemic oppressive policies remains largely unknown. It is likely a combination of both. Similarly, the scale (i.e., household, neighborhood, and beyond) at which structural inequities might affect the gut microbiota is unclear. Nonetheless, the existing literature demonstrates that the same social inequities that predict disease disparities also predict variation in the gut microbiota. These relationships underscore the likely role of the gut microbiota in mediating socially driven health disparities.

## 3. Why is Exercise so Critical?

Exercise has many health benefits. These benefits apply to people of all ages, races and ethnicities, and sexes. Exercise helps individuals maintain a healthy weight, reduces the risk of depression and a decline in cognitive function and lowers a person’s risk for many diseases, such as CVD and other chronic health diseases [3, 4, 5, 6]. When done regularly, moderate- and vigorous-intensity physical activity strengthens the cardiac myocardium and improves the heart’s ability to distribute blood to the body, thereby reducing CVD risk. Exercise can reduce this risk through a variety of mechanisms including lowering blood pressure, and triglycerides, raising HDL (high-density lipoproteins), decreasing arterial stiffness, reducing the risk of being overweight or obese and maintaining a healthy weight, maintenaining in-range blood glucose and insulin levels, and reducing inflammation [3, 4, 5, 6]. This section of the review will highlight the impacts of exercise on the cardiovascular system and the mechanisms by which this occurs, providing a foundation for which we will later discuss the integrated roles of sex, race/ethnicity, CVD, and gut microbiota.

### 3.1 Impacts of Exercise on the Cardiovascular System

**Fig. 1.**
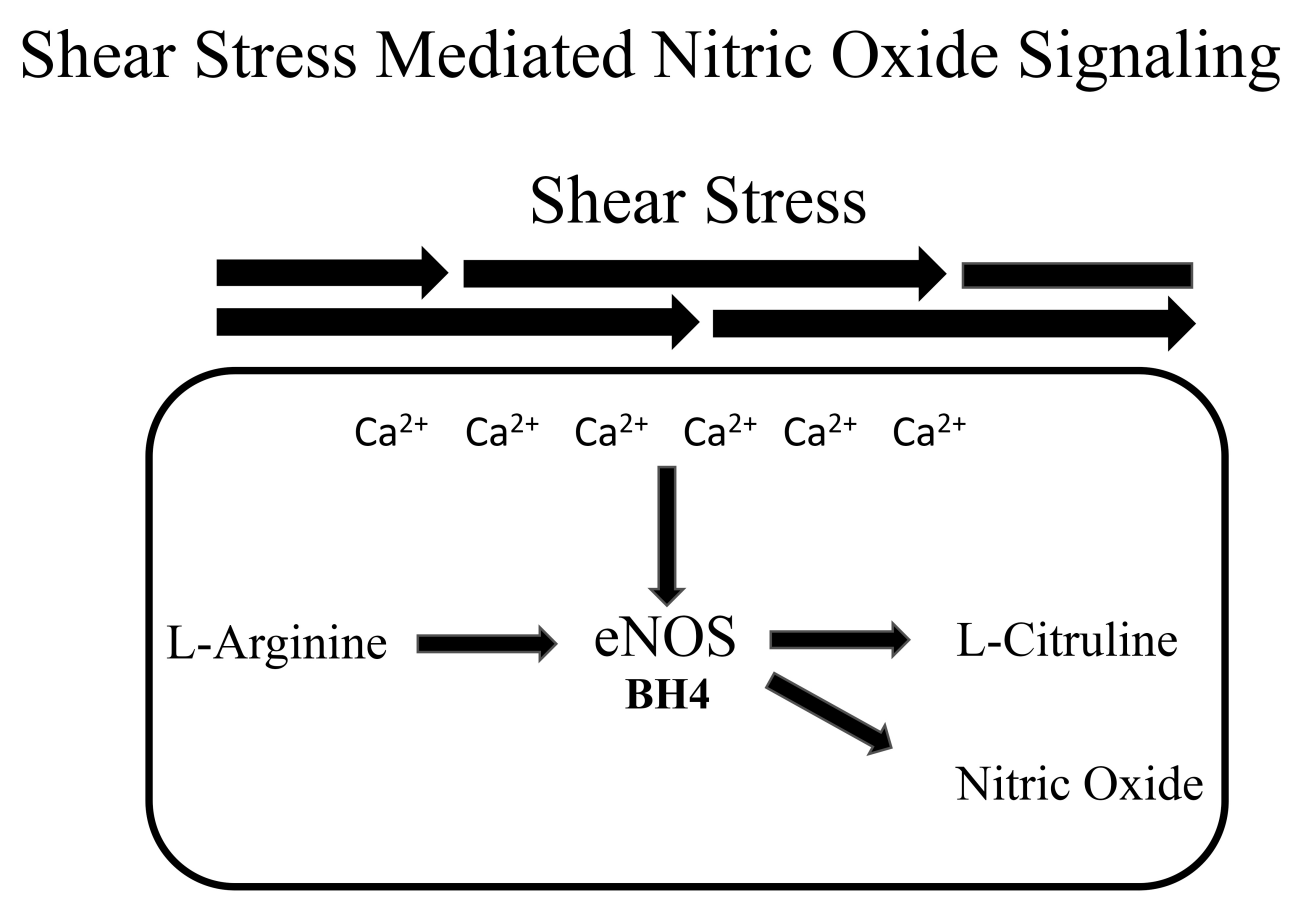
**A representation of nitric oxide signaling**. Shear stress increases intracellular calcium (Ca^2+^) which enhances endothelial nitric oxide synthase (eNOS) enzymatic action. eNOS catalyzes the synthesis of L-arginine to nitric oxide. Tetrahydrobiopterin (BH4) is a critical cofactor.

Broadly, exercise decreases CVD [77] and increased aerobic fitness has been shown to reduce mortality rates of individuals following myocardial infarction [78]. These improvements have been shown in various animal models [79, 80, 81] and human studies [82, 83, 84]. Specifically, it is believed that chronic shear stresses on the endothelial lining of the blood vessels and the endocardium, which are derived from exercise-induced increases in blood flow, increase nitric oxide (NO) bioavailability [85] (Fig. 1). NO is a vasoprotective molecule that prevents vascular dysfunction, platelet aggregation, leukocyte adhesion and vascular stiffening [86, 87]. Reductions in NO have been indicated in the development of hypertension and CVD [88, 89].

Furthermore, exercise augments anti-oxidant defense and decreases reactive oxygen species (ROS) production [90, 91, 92]. Production of ROS is known to increase the potential for cellular damage [93, 94] and can augment the severity of myocardial ischemia [95]. Previous work has shown that exercise-trained rodents have increased cardiac output compared with sedentary littermates following *in-vivo* myocardial ischemia [96]. Exercise has been long known to increase cardiac output via myocardial hypertrophy and proliferation [97]. More recently exercise has been shown to increase peroxisome proliferator-activated receptor-gamma coactivator-1α [98, 99], which has the potential to increase longevity and promote health [100]. Lastly, exercise has several indirect effects that improve cardiovascular health including weight reduction [101] and improved gut health [10]. In the following sections, we will review each of these mechanisms in detail.

### 3.2 Exercise Mediated NO Production

Endothelium-derived NO is essential for cardiovascular health [86, 87] and its production is augmented with acute [102] and chronic exercise [103]. Endothelial-derived NO is synthesized from L-arginine by endothelial nitric oxide synthase (eNOS) and released by endothelial cells [104, 105]. Shear stresses placed on the endothelial cells of blood vessels cause the release of NO, which triggers vasodilation [104, 105]. The repeated shear stresses which are associated with repeated bouts of exercise are thought to increase NO bioavailability by chronically stimulating its release [85].

Improvements in rodent vascular NO bioavailability are often indicated *in-vivo* by examining endothelial-dependent dilation (EDD) in the blood vessel of interest [90]. Because NO is a key regulator of vasodilation, reductions in EDD can be indicative of diminished NO bioavailability. Rodent exercise perturbations ranging from 2–13 weeks have been shown to improve EDD [90, 103, 106] and thus NO bioavailability. This was confirmed in an acute study consisting of two to four weeks of treadmill training in healthy rats. Dose-dependent EDD was improved in the skeletal muscle arterioles of the exercise-trained rats [107], while endothelial independent dilation was not changed. In a 13-week exercise intervention, EDD and NO production in the femoral artery were increased in Wistar-Kyoto rats following treadmill training [108]. Both eNOS expression and phosphorylated eNOS (Ser1177) expression were increased in trained rats when compared to their sedentary littermates.

Exercise also has a vascular protective effect in several models of rodent vascular dysfunction. In a study by Guers *et al*. [109], 6 weeks of voluntary wheel running protected against salt-induced (4% NaCl chow) losses in EDD in rat femoral arteries. Western blot analysis demonstrated that this may have been mediated through a decrease in protein concentration of the reactive oxygen species: nicotinamide adenine dinucleotide phosphate (NADPH) oxidase 4 (NOX4) and Gp91-phox, two subunits of NADPH oxidase. Protein concentrations of both NOX4 and Gp91-phox were initially increased following 6-weeks of a high salt diet in rodents. Exercise also led to an upregulation of the antioxidant superoxide dismutase-2 (SOD2). Collectively, there was a reduction in overall oxidative stress and thus an increase in vascular eNOS bioavailability. eNOS tends to become uncoupled with high levels of oxidative stress [110] and thus becomes unable to synthesize NO [111].

Exercise not only augments NO production in blood vessels but also in the heart [112]. In a study by Kuczmarski *et al*. [113], 4 weeks of voluntary wheel running helped maintain left ventricular (LV) cardiac function following an ischemia-perfusion injury in rats in a model of chronic kidney disease. Kuczmarski found that wheel running protected against losses in LV NO levels and improved overall cardiac redox status [113]. Specifically, this appeared to be mediated through an upregulation of the antioxidant SOD2 [113]. Furthermore, similar to blood vessels, eNOS is upregulated in the heart with chronic aerobic exercise [112]. Dogs who were treadmill trained for 10 days experienced increases in dose dependent EDD in both coronary arteries and the microvasculature of the heart [114]. The authors also found an increase in the constitutive nitric oxide (*ECNOS*) gene. Together these data further support the notion of an increase in NO bioavailability in the heart as a result of exercise.

Exercise also has the potential to increase NO bioavailability in humans [115, 116]. Performing moderate aerobic exercise for 1 hour a day for a month increased NO generation and reduced resting blood pressure. This effect was thought to be mediated through an increase in antioxidant enzymes in blood monocytes [115]. In another study by Tanaka *et al*. [117], the authors discovered that individuals who have high levels of aerobic fitness do not experience the typical age-related decreases in vascular function as measured by EDD. Furthermore, 12 weeks of brisk walking restored losses in EDD in previously sedentary middle-aged and old individuals [117]. Lastly, four weeks of home-based exercise restored losses in forearm EDD in individuals with hypercholesterolemia independent of dietary modifications [118].

### 3.3 Exercise and Heart Failure

Collectively, patients with heart failure tend to have a significant reduction in aerobic capacity [119]. This appears to be at least partially mediated through a reduction in NO [120]. Heart failure patients also consistently have a reduction in EDD [121] which can be partially restored with supplementation of L-Arginine, a precursor of NO [122]. A hallmark of heart failure tends to be the reduction in blood flow back towards the heart which diminishes pre-load. Exercise training has been shown to improve outcomes in patients with heart failure by increasing NO bioavailability and in turn blood flow and preload. Further to this, 12 weeks of aerobic exercise training increases forearm EDD in hypertensive individuals [123].

In both the heart and blood vessels, as indicated in the aforementioned studies, oxidative stress appears to be one of the principal mediators in reducing NO levels consequently disrupting cardiovascular homeostasis. Oxidative stress is defined as an imbalance of free radical production and the production of free radical scavenging antioxidants [124]. Oxidative stress has been indicated in a number of pathologies including CVD [95, 123, 125]. As an example of this: NADPH oxidases were found to be significantly upregulated in aortic atherosclerotic lesions taken from human autopsies [126]. Furthermore, SOD2 knockout mice experienced increased mitochondrial oxidative stress which led to the onset of hypertension [127] and elevations in oxidative stress levels were associated with the severity of heart failure in both the left and right ventricles of mice following myocardial ischemia [128]. Lastly, a clinical studyhas found correlations between markers of oxidative stress and instances of heart failure [129]. Interestingly, in many cases exogenous antioxidants have been shown to improve outcomes in certain instances of CVD [130, 131].

### 3.4 Upregulation of Endogenous Antioxidant Defense

**Fig. 2.**
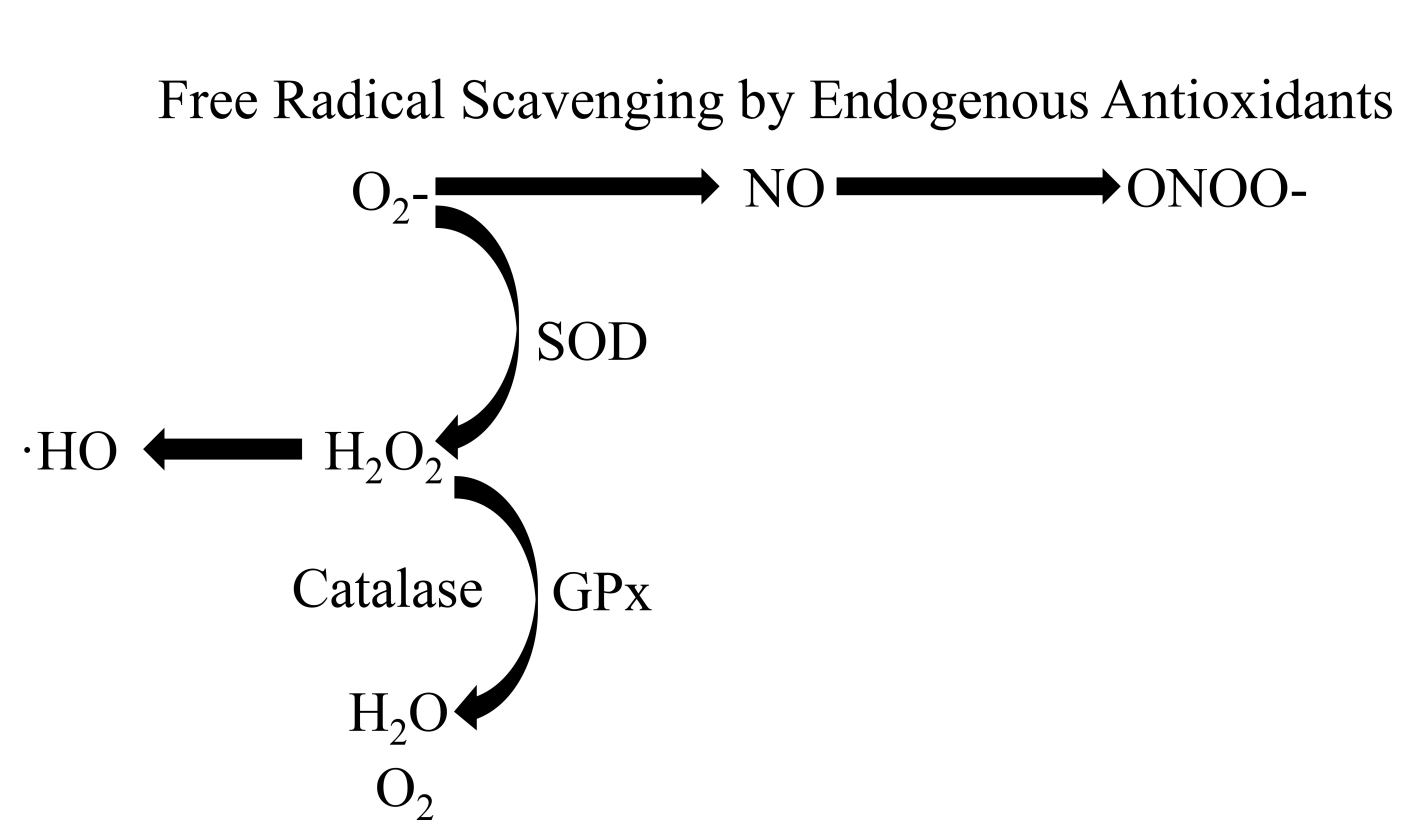
**A representation of free radicals being scavenged by endogenous antioxidants**. Superoxide (O_2_-) reacts with nitric oxide (NO) to form peroxynitrate (ONOO-). Superoxide dismutase (SOD) catalyzes the reaction of O_2_- to hydrogen peroxide (H_2_O_2_), which participates in the formation of hydroxyl radicals (⋅HO). Both catalase and glutathione peroxidase (GPx) reduce H_2_O_2_ to water (H_2_O) and oxygen (O_2_).

As mentioned earlier exercise has the capacity to increase antioxidant defenses and decrease oxidative stress levels which protects against a reduction in NO bioavailability and maintains normal cardiovascular function. SOD is an antioxidant that can be upregulated through exercise [109, 113]. SOD is critical in the maintenance of cardiovascular homeostasis as it prevents the breakdown of NO by the reactive oxygen species superoxide (O_2_^.-^) [132]. O_2_^.-^ has a high affinity for NO and rapidly converts it to peroxynitrite (ONOO-) which can damage lipoproteins. SOD reacts and dismutates O_2_^.-^ to H_2_O_2_ before this reaction can occur. An increase in O_2_^.-^ disrupts vascular function [133] and elevations in ONOO- levels are associated with CVD [134] (Fig. 2).

Therefore, a deficiency in SOD will lead to a decrease in NO bioavailability and diminishes vascular function. As an example, copper zinc SOD (CuZnSOD) deficient mice had a 2-fold increase in O_2_^.-^ relative to their control littermates. Ultimately, this led to a decrease in dose-dependent EDD in the carotid artery [135]. Reduced SOD has also been associated with a number of pathologies including atherosclerosis, hypertension, and hypercholesterolemia [136]. Importantly, as mentioned previously aerobic exercise can increase SOD levels. In a study by Durrant *et al*. (2009) [103], old mice with access to a running wheel had greater levels of aortic SOD and lower levels of NADPH oxidases relative to their untrained littermates. This coincided with better dose-dependent EDD and higher levels of aortic eNOS and phosphorylated eNOS (Ser1177) expression [103].

H_2_O_2_, is the result of the dismutation of O_2_^.-^ by SOD and elevated levels of H_2_O_2_ can also lead to oxidative stress [93] and vascular dysfunction [137]. The antioxidants, Glutathione peroxidase (GPx) and catalase are both capable of reducing H_2_O_2_ to oxygen and water. In humans, low levels of GPx are associated with an increased risk of CVD [138]. Furthermore, in mice, GPx deficiency led to a reduction in NO and a decrease in vascular function [139]. Similar to GPx, low levels of catalase are also associated with CVD [140]. Like SOD, several studies have shown that exercise increases levels of both GPx and catalase [141].

### 3.5 Protection from Arterial Stiffness

Arterial stiffness is a consistent independent predictor of all-cause mortality in individuals with hypertension [142]. Arterial stiffening is often associated with atherosclerosis, aging, smoking, obesity, and hyperlipidemia amongst other factors [143]. Over time, the structural properties of the vasculature can change. Collagen deposition in the tunica media and the degradation of elastin decreases the ability of arteries to dampen pulse waves and increases blood pressure [144]. Furthermore, chronic elevations in blood pressure increase LV overload which leads to the eventual development of LVventricular hypertrophy. Specifically, the loss of the ability to “dampen” a pulse wave in the aorta leaves organs with low vascular resistance vulnerable to injury [144]. One particular example is the kidneys where the exacerbation of damage is associated with the stiffening of both resistance arteries as well larger elastic arteries [145].

It has been established that exercise has the ability to slow down and help prevent vascular stiffening as well as decrease collagen levels in rodents [90, 91, 146] and humans [147, 148]. Further, arterial stiffness tends to be correlated with maximal aerobic capacity [130]. Fleenor *et al*. 2010 [146], found that 10–14 weeks of voluntary exercise was associated with decreased age-related vascular stiffness. Specifically, collagen I and III fibers were reduced. Another study examining a model of heart failure in mice discovered that 6 weeks of treadmill exercise was able to prevent the onset of aortic stiffening relative to sedentary mice [149]. Wheel running also protected young and old mice from arterial stiffness after consuming a Western-style diet (40% fat and 19% sucrose) for 10–14 weeks. Sedentary mice placed on a Western-style diet also had diminished EDD and NO bioavailability, exercise protected from losses in both. Lastly, rats placed on a high salt diet for 6 weeks experienced increased vascular stiffness and aortic collagen I protein expression [90]. All of these variables were attenuated when rats were given access to a running wheel during the same 6 weeks. Exercise-trained mice also had higher levels of aortic SOD2 protein expression when compared to sedentary rats who were placed on the same diet.

Arterial stiffening and oxidative stress tend to go hand in hand. Oxidative stress is a known initiator of vascular inflammation [150]. Studies have shown that antioxidant therapy is successful at decreasing oxidative stress and arterial stiffness. While this appears evident in animal models [131, 151] the results tend to be mixed in human trials [150, 152]. When TEMPOL (4-hydroxy-2,2,6,6-tetramethylpiperidin-1-oxyl), a superoxide dismutase mimetic was given to aging mice, not only was EDD improved there was lower levels of oxidative stress and large artery stiffness decreased [131]. Mitoquinone (MitoQ), an antioxidant which targets mitochondrial specific reactive oxygen species, not only reduced oxidative stress in aging mice but decreased aortic stiffness [153]. MitoQ was also shown to be effective in healthy older adults. Following chronic supplementation brachial flow-mediated dilation and aortic stiffness were lower [151].

Therefore, reduction and protection from arterial stiffness may be related to the ability of exercise to reduce oxidative stress. Spontaneous hypertensive rats had reduced vascular stiffness in the mesenteric and coronary arteries following 12 weeks of treadmill training. Authors found that these mice also had high NO bioavailability and less evidence of oxidative stress when compared to the spontaneous hypertensive rats who did not exercise [92]. Finally, voluntary wheel running reversed aortic stiffening in old mice. There was also a subsequent reduction in aortic O_2_ bioavailability [154].

## 4. Getting to the Heart of the Matter

### 4.1 The Gut-Heart Axis and CVD: An Update

We have previously reviewed the strong connection between the gut microbiome and cardiovascular disease, showing how dysbiosis and specific gut-derived metabolites can cause endothelial dysfunction, large artery stiffening, hypertension, and ultimately CVD [155]. Since our review on this topic, the literature has continued to evolve and continues to support a strong association between the gut microbiome and CVD. Here, we will summarize seminal new findings on the gut-heart axis since the publication of our previous review.

Studies since our last review have focused on understanding the role of gut microbial derived metabolites in CVD [156, 157, 158]. These studies have produced equivocal results with some metabolites like Indole-3-Propionic acid protecting against heart failure in patients with preserved ejection fraction [159], but others like butyrate showing no signs of altering, perhaps increasing CVD related diseases like hypertension [160] while gut microbial metabolite imidazole propionate (ImP) is increased in individuals with heart failure and is a predictor of overall survival [161].

With regards to studies associating specific gut microbiota to CVD, there have been some recent advances. Okami *et al*. [162], showed that as coronary artery calcification (CAC) scores rose in Japanese men, so did the Bacillota to Bacteroidota ratio, suggesting a relationship between higher gram-positive microbes and artery calcification. Given this is at such a high level of taxonomic resolution, the authors further reported that *Lactobacillales* were associated with a 1.3- to 1.4-fold higher risk of CVD and a higher CAC score. In addition, presence of *Streptococcaceae* and *Streptococcus* were linked to a higher risk of CVD while *Enterobacteriaceae* correlated with CAC scores. Sayols-Baixeras *et al*. [163], showed that *Streptococcus anginosus* and *Streptococcus oralis* had the strongest associations to CAC. Keeping in mind findings at the level of species and strain could be beneficial for the generation of -biotics, using bugs and drugs. Salvado *et al*. [164], showed that early vascular aging was associated with *Bilophila*, *Faecalibacterium sp.UBA1819* and *Phocea*. Furthermore, when logistic regression analysis was completed, *Bilophila* remained significant. This is important because animal work has shown that *Bilophila. wadsworthia* caused systemic inflammation, suggesting the pathogenicity of this bacterium [165]. Guo *et al*. [166], showed that the genera *Escherichia-Shigella*, *Lactobacillus*, *Enterococcus* were more abundant in patients with resistant hypertension compared to normotensive adults.

While trimethylamine N-oxide (TMAO) continues to be a major gut microbial-derived metabolite of focus for CVD [167], an emerging metabolite phenylacetylgutamine (PAGln) has received a lot of attention recently [168]. In 2020, PAGln was discovered and is both associated with atherothrombotic heart disease in humans [169, 170, 171], and mechanistically linked to cardiovascular disease pathogenesis in animal models via modulation of adrenergic receptor signaling [172, 173]. Since then, Romano *et al*. [174] demonstrated that circulating PAGln levels were dose-dependently associated with heart failure presence and indices of severity (reduced ventricular ejection fraction, elevated N-terminal pro-B-type natriuretic peptide) independent of traditional risk factors and renal function, with associations between TMAO and incident heart failure being stronger among Black and Hispanic/Latino adults compared to White adults. Similar findings were shown by Tang *et al*. [175], which extended the work to show that PAGIn levels, independent of TMAO, may be used as a predictor of future CV events.

Despite these recent advances, mechanistic studies are still either in their infancy or lacking in the field and even more importantly studies which compare sex and race/ethnicity need urgent attention. Knowledge of which gut microbes may be involved is a good start, but understanding their function and role in the development of CVD is still lacking. Finally, there has been a lot of attention on ways to manipulate the gut microbiota via fecal transplants, symbiotics, probiotics, high-fiber diets and prebiotics, while this is outside the scope of this review, it has recently been reviewed elsewhere and the authors call your attention to Theofilis *et al*. [176].

### 4.2 Racial Variation in CVD

Despite trends for reductions in mortality rates from CVD in the US between 1980 and 2010, deaths attributable to CVD are once again on the rise. One pattern that has remained constant during this time is that racial and ethnic minority groups in the US (and globally) experience a disproportionate burden of CVD compared to their White counterparts [177, 178, 179, 180]. Overall, CVD prevalence remains highest among non-Hispanic Black women (59%) and non-Hispanic Black men (58.9%) [179, 181]. Black women and Black men are more than twice as likely to die of CVD, relative to White women and White men [179, 181] and among young and middle-aged adult survivors of a myocardial infarction, Black patients have a 2-fold higher risk of adverse outcomes [182].

It has been suggested that hypertensive target organ damage is widespread in Black and African American adults [183]. Young Black patients have an increasing burden of CVD risk factors [177]. Individuals of Black and African American ancestry experience hypertensive target organ damage earlier in life compared with White Americans [184]. Black/African American adults may also be more susceptible to the damaging effects of high blood pressure [185, 186]. Numerous studies note large disparities in measures of vascular health, with Black/African American adults displaying lower NO-mediated EDD and higher large artery stiffness and pressure from wave reflections compared with White Americans [187, 188, 189]. We and others have shown that disparities in these vascular health measures can be seen in childhood and correlate with proxies of target organ damage such as carotid intima-media thickness, LV mass, myocardial work, and coronary perfusion [190, 191, 192, 193]. Such “early vascular aging” in Black/African American adults likely serves as the catalyst for detrimental LV remodeling, heart failure, and future CVD [194]. For the past several decades, racial differences in CVD were ascribed to biological (“genetic”) differences (e.g., biological differences in inflammation, oxidative stress, NO metabolism, renin-angiotensin-aldosterone system, and autonomic nervous system function), neglecting the crucial role of the environment on risk [195, 196, 197]. It is now commonly recognized that cardiovascular health disparities are driven largely by deep-rooted structural racism and not race per se [178, 198, 199, 200].

Individuals who self-identify as members of a racial or ethnic minority group experience greater obstacles to health due to social, economic, and/or environmental disadvantages [199]. Systemic oppressive structures, policies, and practices in the US (i.e., social injustice) have created inequity in access to resources, services, and opportunities in minoritized (and marginalized) groups, driving disparities in SES and cardiovascular health [201]. Minority-related psychosocial stressors experienced by marginalized groups such as prejudice, discrimination, pressure to conform to a group stereotype by members of the same marginalized group, and pressure to acculturate/acculturation, are emerging as powerful risk factors for CVD and cardiovascular mortality [202]. Indeed, perceived discrimination is associated with increased risk for hypertension, systemic inflammation and oxidative stress, subclinical atherosclerosis, and detrimental vascular remodeling (increased carotid intima-media thickness, coronary artery calcification, and large artery stiffness), target organ damage, myocardial infarction, heart failure, and stroke [203, 204, 205, 206, 207]. Other factors related to structural racism such as lower SES, educational attainment, place of birth, neighborhood safety and food insecurity from residential segregation, and built environment (i.e., access to blue and green space, also shaped by neighborhood-level racial residential segregation) are barriers to ideal cardiovascular health [208, 209, 210, 211, 212, 213]. Moreover, each of these social determinants of health (SDoH) along with others such as stress from the incarceration of family or friends, job insecurity, violence in the home setting, and healthcare access are also associated with hypertension, inflammation, and oxidative stress, subclinical atherosclerosis, detrimental vascular remodeling, target organ damage, and ultimately CVD [214, 215, 216, 217, 218, 219, 220, 221]. We have recently shown that environmental toxicants found in higher concentrations in areas of lower SES are “cardiovascular disruptors” in children, contributing to altered vascular reactivity (greater blood pressure and vascular resistance in response to psychological stress) and subclinical CVD measured as carotid intima-media thickness at a young age [222, 223, 224, 225]. Additionally, we have shown that relative to White children, Black children have significantly greater hair cortisol levels and flatter diurnal slopes, which were in turn associated with subclinical CVD (measured as carotid intima-media thickness and aortic stiffness) [222]. Black children experienced significantly more environmental stress than White children with income inequality partially explaining the higher subclinical CVD risk in Black children [222]. Taken together, psychosocial determinants are the likely drivers of early (premature) vascular aging in Black and African American people in the US, some of which may be transmitted intergenerationally via biological (i.e., prenatal fetal programming) and social (i.e., early life adversity) mechanisms. This hypothesis is in keeping with minority stress theory and the weathering hypothesis whereby chronic exposure to social and economic disadvantage leads to increased allostatic load and accelerated biological (and physiological) “wear and tear” on end organs causing inflammation and oxidative stress, hastening aging [226].

### 4.3 Racial Variation in the Gut-Heart Axis: Implications for CVD

This section will examine racial variation in the gut microbiome with consideration for how the systemic environment (i.e., structural racism) impacts the microbial environment to perpetuate cardiovascular health disparities. As introduced above, there is growing evidence that the social and environmental gradients which contribute to health inequities also predict gut microbiota traits [227]. Evidence shows that the human microbiome variation is linked to the incidence, prevalence, and mortality of many diseases and is associated with race and ethnicity in the US. To date, there have been several studies (discussed next) that have examined this outcome and have identified gut microbiota profiles shaped by host environments which affect host metabolic, immune, and neuroendocrine functions, making it an important pathway by which differences in experiences caused by social, political, and economic forces could contribute to health inequities.

It is thought that the gut microbiota is well established by the time a child is 4 years old, and there is strong evidence that maternal, and family socioeconomic status can influence gut microbiota. Several investigators have analyzed data from the Food and Microbiome Longitudinal Investigation (FAMiLI) study to obtain answers on how maternal family and SES influences the gut. FAMiLI is an ongoing multi-ethnic prospective study in the US that began in 2016 where participants complete demographic questionnaires and (optional) food frequency questionnaires and provide oral and stool samples. In 2020, Peters *et al*. [228], analyzed samples from 863 US residents, including US-born (315 White, 93 Black, 40 Hispanic) and foreign-born (105 Hispanic, 264 Korean). The authors determined dietary acculturation from dissimilarities based on food frequency questionnaires and used 16S rRNA gene sequencing to characterize the microbiome [228]. Their results showed a clear difference in gut microbiome composition across study groups. They found the largest differences in gut microbiota between foreign-born Koreans and US-born Whites, and significant differences were also observed between foreign-born and US-born Hispanics. Specifically, differences in sub-operational taxonomic unit (s-OTU) abundance between foreign-born and US-born groups tended to be distinct from differences between US-born groups. *Bacteroides plebeius*, a seaweed-degrading bacterium, was strongly enriched in foreign-born Koreans, while *Prevotella copri* and *Bifidobacterium adolescentis* were strongly enriched in foreign-born Koreans and Hispanics, compared with US-born Whites. Dietary acculturation in foreign-born participants was associated with specific s-OTUs, resembling abundance in US-born Whites; e.g., a *Bacteroides plebeius* s-OTU was depleted in highly diet-acculturated Koreans. The authors concluded that US nativity is a determinant of the gut microbiome in a US resident population.

The “sociobiome” was coined by Nobre and Alpuim Costa [229] to describe the microbiota composition occurring in residents of a neighborhood or geographic region due to similar socioeconomic exposures; socioeconomic status. Recently, Kwak *et al*. [230], using the FAMiLI cohort, investigated the sociobiome in a large, multi-ethnic sample. The cohort consisted of 825 adults (36.7% male), with a mean age of 59.6 years and racial and ethnic group composition consisting of 311 (37.7%) non-Hispanic White, 287 (34.8%) non-Hispanic Asian, 89 (10.8%) non-Hispanic Black, and 138 (16.7%) Hispanic participants and compared alpha-diversity, beta-diversity, and taxonomic and functional pathway abundance by SES. They showed that lower SES was significantly associated with greater α-diversity and compositional differences among groups, as measured by β-diversity. Several taxa related to low SES were identified, especially an increasing abundance of *Prevotella copri* and *Catenibacterium sp000437715*, and decreasing abundance of *Dysosmobacter welbionis* in terms of their high log-fold change differences. This is significant as *Dysosmobacter welbionis* was isolated from human commensal bacterium from samples provided by the Human Microbiome Project, American Gut Project, Flemish Gut Flora Project and Microbes4U projects. This bacterium was detected in 62.7%–69.8% of the healthy population and correlates negatively with body mass index, fasting glucose and glycated hemoglobin. In addition, Cani’s group using the humanized mouse model, taking human fecal samples/strains and putting them into a mouse, showed that *Dysosmobacter welbionis* prevented diet-induced obesity and metabolic disorders in mice by reducing fat mass gain, insulin resistance and white adipose tissue hypertrophy and inflammation [231]. In addition, live *Dysosmobacter welbionis* administration protected the mice from brown adipose tissue inflammation in association with increased mitochondria number and non-shivering thermogenesis. While this has yet to be translated to humans, the reduction of this bacteria in the human study coupled with its actions seen in animal studies suggest that the lack of this bacteria may place individuals at increased risk for metabolic disorders and adipose tissue dysfunction which could lead to adverse CVD outcomes.

Most recently, Mallott *et al*. [232], set out to determine the age at which microbiome variability emerges between race and ethnic groups. They used 8 datasets with 16S ribosomal RNA (rRNA) sequencing data and available race and ethnicity metadata for this study. Individuals between birth and 12 years of age, living in the US, with a caregiver-reported race of Black, White, or Asian/Pacific Islander, and with a caregiver-reported ethnicity of Hispanic or non-Hispanic were included in the analysis. They found that race and ethnicity did not significantly vary with gut microbiome alpha-diversity or beta-diversity in the early weeks and months of life, including the first week, 1 to 5.9 weeks, and 6 weeks to 2.9 months, however, at 3 to 11.9 and 12 to 35.9 months, gut microbiome composition varied slightly but significantly by both race and ethnicity. The group concluded that race and ethnicity are associated with gut microbiome composition and diversity beginning at 3 months of age, indicative of a narrow window of time when this variation emerges [232].

Finally, discrimination and stress have been found to contribute to changes in gut microbiota among racial and ethnic groups [233, 234]. A study by Dong *et al*. [235], examined 154 adults from the Los Angeles community and clinics. Participants self-reported race and ethnicity (Asian American, Black, Hispanic, or White) and discrimination was measured using the Everyday Discrimination Scale. Hispanic individuals self-reported the highest levels of early-life adversity, while Black individuals reported the highest levels of resilience. Microbiome and metabolite differences related to discrimination were only apparent when stratified by race/ethnicity. Results showed that *Prevotella copri* was the highest in Black and Hispanic individuals, who experienced high levels of discrimination, whereas White individuals reported low levels of discrimination. Isovalerate and valerate were significantly lower in Hispanic than in White individuals and fucosterol was significantly higher in Asian rather than White individuals. In a related study, Zhang *et al*. [236], investigated the impact of discrimination exposure on brain reactivity to food images and associated dysregulations in the brain–gut–microbiome axis. By employing multi-omics analyses of neuroimaging and fecal metabolite, they showed that discrimination is associated with increased food-cue reactivity in regions of the brain important for reward, motivation and executive control; altered glutamate-pathway metabolites involved in oxidative stress and inflammation as well as a preference for unhealthy foods. In addition, the relationship between discrimination-related brain and gut signatures was shifted towards unhealthy sweet foods after adjusting for age, diet, body mass index, race and SES. Given the extensive literature on diet, obesity and the gut microbiota, these results are significant in suggesting that individuals facing discrimination may prefer unhealthy foods (and/or may not have access to healthy foods) contributing to a more dysbiotic gut and thus adverse cardiometabolic health outcomes.

**Fig. 3.**
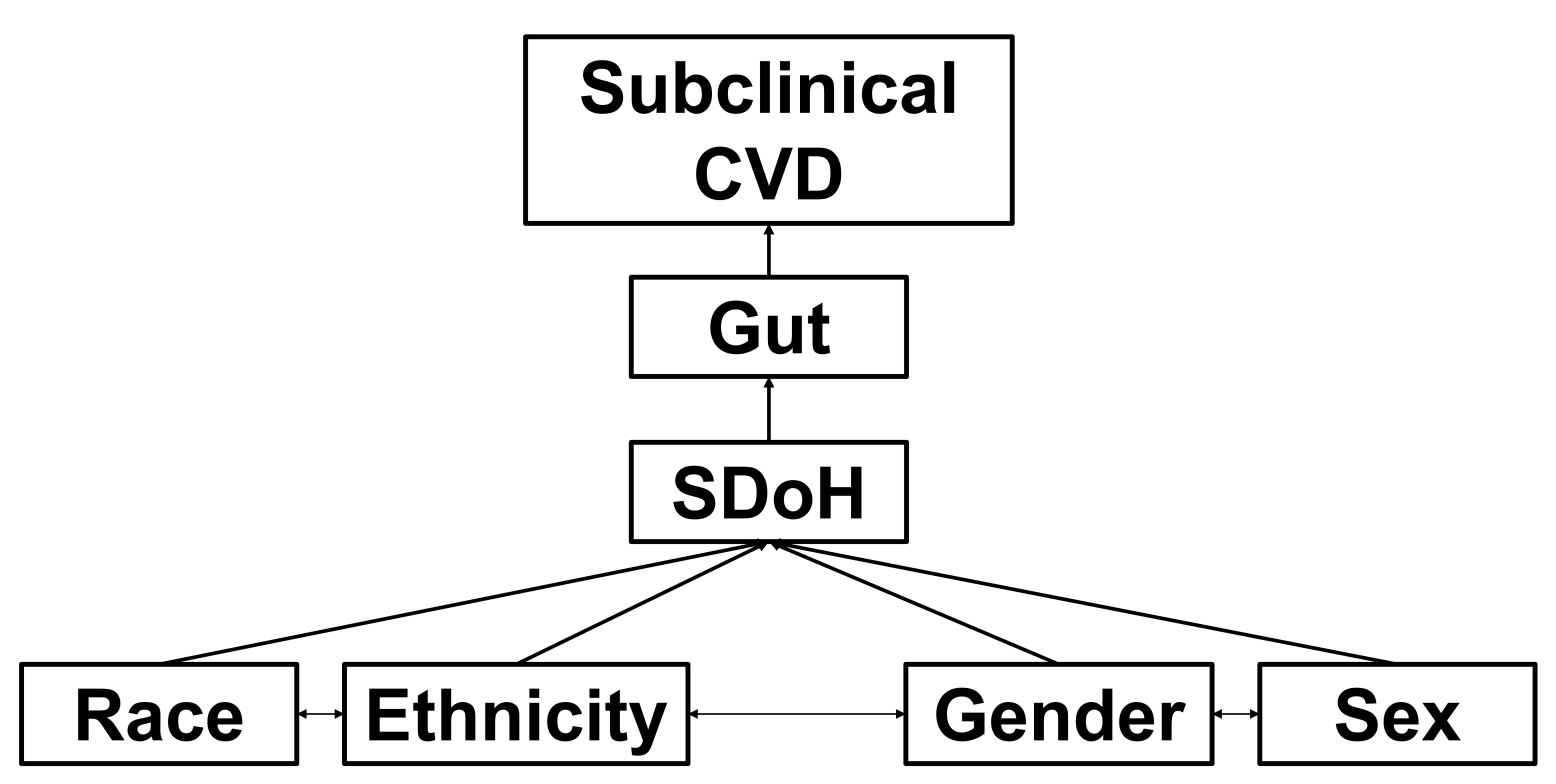
**Working conceptual model**. Race, ethnicity, gender, and sex interact (i.e., intersectionality) and are shaped by social determinants of health (SDoH) to moderate gut effects (dysbiosis, diversity, specific metabolites, gut “age”) on subclinical cardiovascular disease (CVD) (endothelial dysfunction, large artery stiffness) - driving CV health disparities and overt CVD (hypertension, coronary ischemia and vasospasm, myocardial infarction, heart failure). CV, cardiovascular.

In conclusion, there are distinct gut microbiota profiles between racial and ethnic groups, which appear to be influenced by acculturation [237, 238, 239], discrimination and stress [233, 234], and diet [240], which may occur as early as 3 months of age. Where a person lives and the related neighborhood and environmental constraints, what stresses they are exposed to, and what a person eats (both what they choose to eat and what they have access to eat) may shape the gut microbiome more than race or ethnicity per se. Finally, these distinct gut microbial community structures can exacerbate CVD risk among minority racial and ethnic groups [241] (Fig. 3).

### 4.4 Biological Sex, Gender and CVD

Another prejudice that has a profound impact on health and CVD risk is sexism [242]. Women, in general, have also been historically marginalized due to institutionalized patriarchy and a male-dominated social system. When considering the impact of sexism on CVD, we must first operationalize and contextualize differences (and overlap) between biological sex and gender. Sex, when considered biologically, comprises genetic differences related to chromosomes, gonadal structure and function, and hormonal sequela. We will conceptualize sex as referring to male, female, and intersex. Gender is a social construct based on sociocultural predetermined roles, relationships, and stereotypes (e.g., masculine versus feminine). Gender can be shaped by different power dynamics and how we interact with others around us based on ascribed gender and can vary based on regionality, nationality, and temporality (i.e., ideals can change over time). Gender also encompasses gender identity referring to a person’s inner sense of self as a man, woman, nonbinary person, or agender person among other identities. Sex and gender can be considered together to inform on both biological sex and self-identified gender. For example, a person who identifies as a cis-gender woman is a woman whose self-identified gender aligns with the biological sex assigned at birth.

In the context of CVD, biological sex and gender may converge to affect risk [243, 244]. Women are typically believed to be at lower risk for CVD owing to the biological effects of the gonadal hormone estrogen. Note here that we do not consider estrogen a sex hormone per se as both men and women produce estrogen (and testosterone), just in varying amounts. Just as low estrogen is associated with increased risk for coronary heart disease and CVD mortality in older men [245], low testosterone is associated with a greater risk of ischemic CVD and major adverse cardiovascular events in older women [246, 247]. Subsequently, CVD risk increases in women with advancing age, particularly post-menopause. With that said, it should be highlighted that CVD remains the leading cause of mortality in women of all ages, and hospitalizations and deaths attributed to CVD have witnessed an increase for younger and middle-aged women [248]. The reasons for these observations are likely multifactorial and may partly be related to societal sex- and gender-based discriminatory attitudes [249]. Not until the American Heart Association’s “Go Red” campaign has there been equitable education and promotion of CVD risk for women. As such, educational efforts on signs, symptoms, risk factors, and consequences of CVD in women were sparse. This may have contributed to increased CVD risk factor burden in women and women being less likely to seek timely medical care for signs and symptoms related to CVD. As cardiology is still a predominantly male workforce drawing from scientific literature where women are underrepresented, implicit bias may affect clinical decision-making. For example, signs of myocardial infarction are often categorized as “atypical” in women not because they are abnormal but because they are different from men, with male symptomology being construed as the norm. Some male physicians may also incorrectly assume that a younger/middle-aged woman presenting with chest pain cannot be having a myocardial infarction because that would go against the entrenched dogma that estrogen is cardioprotective. As a result, when seeking care, women have longer wait times when presenting with chest pain, are more likely to be misdiagnosed, more likely to have symptomology dismissed, and are less likely to be prescribed medications or treatments known to mitigate risk [250]. Women are also less likely to be referred to cardiac rehabilitation after a cardiac event [251, 252]. Together, all of these factors contribute to women having poorer outcomes after a cardiovascular event compared to men.

Women are more likely to develop concentric LV remodeling and heart failure with preserved ejection fraction than men [253]. The pathophysiology of coronary artery disease also differs by sex with women possibly having coronary endothelial dysfunction and microvascular defects compared to men, contributing to sexual dimorphism in acute coronary syndromes [254]. While premenopausal women may have better endothelial function than men [255], we and others have shown that women may have greater pressure from wave reflections increasing central hemodynamic load [256, 257, 258]. Sex differences in central hemodynamic burden may contribute to greater LV diastolic dysfunction and associations between arterial stiffness and LV mass/LV diastolic dysfunction may be greater in women compared to men [259, 260, 261]. Large artery stiffness increases disproportionately in postmenopausal women and the association between large artery stiffness and CVD mortality is almost twofold higher in women versus men [262]. As noted above, it is difficult to parse out how much CVD risk is attributable to sex and how much to gender. Some CVD risk in this setting has been suggested to be related to stature (e.g., smaller coronary arteries experiencing more shear stress, shorter aortic length contributing to greater pressure from wave reflections) [263, 264], which may be theorized to be biologically driven. Some CVD risk may be related to the physiological response to mental stress [265, 266, 267], which may be influenced by psychosocial determinants of health. Myocardial ischemia and peripheral microvascular endothelial dysfunction in response to mental stress are greater in women compared to men and associated with major adverse cardiovascular events in women only [268]. Taken together, CVD risk in women likely captures the interaction of both sex and gender on cardiovascular structure and function.

While traditional risk factors (age, lipids, glucose, smoking, blood pressure) affect CVD risk in women and men similarly, there are also sex-specific risk factors that are critically important to consider for women [269]. Sex-specific risk factors relate to biological variation in reproductive health factors and are uniquely ascribed to female biological sex [270]. Such risk factors may include adverse pregnancy outcomes (e.g., hypertensive disorders of pregnancy, gestational diabetes, fetal growth restriction, preterm delivery, and placental abruption), premature menarche, premature menopause and vasomotor symptoms, endometriosis and polycystic ovarian syndrome [270]. Additionally, there are other emerging CVD risk factors caused by other comorbidities and social factors that are more prevalent in women and may be influenced by both sex and gender. These factors include autoimmune disorders, migraine, fibromyalgia, postural orthostatic tachycardia syndrome, osteoporosis, breast cancer, irritable bowel syndrome, abuse, intimate partner violence, post-traumatic stress disorder, anxiety, and depression [270]. Each of the aforementioned female sex-specific and female sex-prevalent risk factors is associated with increased risk for hypertension, systemic inflammation and oxidative stress, subclinical atherosclerosis, and detrimental vascular remodeling (increased carotid intima-media thickness, coronary artery calcification, and large artery stiffness), target organ damage, myocardial infarction, heart failure, and stroke [271].

When considering intersectionality, Black and Hispanic women may encounter “double jeopardy” due to the combination of race and ethnicity bias, coupled with sex and gender bias [272]. Minority women experience additional ethnic, racial and gender constraints and risks including reduced health care access, possible language barriers, lower health literacy, racial discrimination, pressure to acculturate or conform to both a racial and culturally gendered identity, higher reports of depression and higher incidence of pregnancy complications (e.g., hypertensive disorders of pregnancy) [273, 274]. As stated above, these SDoH are also CVD risk factors and are as important and sometimes more important correlates of subclinical CVD in women [275, 276, 277, 278, 279, 280, 281]. As such, the prevalence of sex-specific CVD risk factors, coronary artery disease, heart failure, and stroke is highest among non-Hispanic Black women [282]. As stated by the American Heart Association, to understand and address the root causes of the prominent disparities in CVD outcomes between Black and White women and men in the United States, the intersectional aspects between race, sex, and gender must be considered [283]. Nearly 60% of Black women have CVD, contributing to a persistent life expectancy gap in the US [181]. Current life expectancy for Non-Hispanic Black women is 75 years on average compared with 80 years for non-Hispanic White women [269]. CVD is also the most prominent cause of mortality amongst Hispanic women, with approximately 42% of Hispanic women having CVD [181]. Paradoxically, despite a higher prevalence of such traditional CVD risk factors such as diabetes, obesity, and metabolic syndrome, CVD death rates in Hispanic women have remained 15% to 20% lower than in non-Hispanic White women - an observation commonly referred to as the Hispanic Paradox [284]. Interestingly, we have seen that young Hispanic women have better endothelial function and lower large artery stiffness compared to White women [285], suggesting that traditional CVD risk factors may not capture actual CVD risk in this population. It should be noted that this paradox is disappearing as Hispanic American individuals acculturate and adopt the high-fat, sedentary lifestyle of those with US nativity [286]. As noted above, sex differences in the vascular response to mental stress are a predictor of major adverse cardiovascular events in women. Endothelial dysfunction in response to mental stress is also a predictor of adverse CV outcomes in Black adults, explaining 69% of their excess risk [287]. Notable predictors of the development of transient endothelial dysfunction with mental stress beyond Black race include female gender, employment status, income, and a composite distress score derived from post-traumatic stress disorder, depression, anxiety, anger, perceived stress and racial discrimination [288, 289, 290, 291]. These findings highlight the importance of intersectionality and psychosocial determinants of vascular health impacting CVD risk in women, particularly Black women.

There is also emerging evidence that lesbian, gay, bisexual, transgender, and queer or questioning (LGBTQ+) adults, as a stigmatized and marginalized group, experience notable cardiovascular health disparities [292, 293]. According to the American Heart Association, people who are transgender and gender diverse may be at greater risk for CVD [294]. There is growing evidence that LGBTQ+ adults experience worse cardiovascular health relative to their cisgender heterosexual peers [292, 295]. For example, men who are transgender have a >2-fold and 4-fold increase in the prevalence of myocardial infarction compared with men who are cisgender and women who are cisgender, respectively. Conversely, women who are transgender have >2-fold increase in the prevalence of myocardial infarction compared with women who are cisgender. Moreover, compared to heterosexuals, sexual minorities are at a higher risk of hypertension and CVD and more likely to develop CVD at an earlier age [296, 297]. It should be underscored that the LGBTQ+ (intersexual, asexual, pansexual, two spirit) community is not a monolithic group [298]. Each has unique lived experiences that may subsequently shape CVD risk. Differences in CVD risk are partially, but not completely, explained by traditional CVD risk factors suggesting that SDoH plays a significant role. LGBTQ+ adults not only experience significantly higher discrimination from the broader community, but also specifically from healthcare professionals [299]. Additional psychosocial risk factors including self-stigma and internalized phobia, gender-related victimization, expectations of rejection, and concealment, all detrimentally impact mental health (anxiety, depression) and behavioral health (diet, sleep, physical activity, alcohol and tobacco/nicotine use) [300, 301]. Together, these factors may contribute to inflammation and oxidative stress, hastened vascular aging, subclinical atherosclerosis, target organ damage and overt CVD [302, 303].

Biological effects of gender-affirming hormone therapy (GAHT) may also have an impact on CVD risk [304, 305]. Use of GAHT in transgender and nonbinary individuals is perceived to improve cardiovascular health [306]. The association between GAHT and CVD risk is complex [307]. A higher blood concentration of testosterone among women who are transgender is associated with higher odds of having hypertension. Cross-sectional comparisons between men who are transgender receiving testosterone cypionate compared with age-matched women who are cisgender have found reduced endothelial function measured via brachial artery flow-mediated dilation [308]. In cross-sectional studies, carotid intima-media thickness, arterial stiffness and measured via brachial-ankle pulse wave velocity, and carotid augmentation index are higher in men transitioning (female to male) receiving testosterone than in men who are transgender not receiving hormone therapy [309, 310, 311]. Similarly, transgender men on long-term treatment with testosterone have higher aging-related aortic stiffening [312], suggesting accelerated vascular aging in transgender men receiving gender-affirming hormone treatment. This is supported by animal studies noting that female mice receiving dihydrotestosterone experience hastened rates of arterial stiffening and cardiovascular damage, mediated by decreased estrogen receptor expression [313]. Brachial artery flow-mediated dilation is higher in women who are transgender treated with estrogen than in age-matched men who are cisgender but is similar to women who are cisgender [314, 315]. Women who are transgender receiving estrogen also have a greater forearm blood flow response to acetylcholine, an endothelial-dependent vasodilator, than age-matched men who are cisgender [314]. In summary, GAHT is associated with an increased risk of subclinical atherosclerosis in transgender men but may have either neutral or beneficial effects in transgender women [316].

### 4.5 Biological Sex, Gender and the Gut-Heart Axis: Implications for CVD

This section will consider the mediating and moderating effects of sex, sex-specific CVD risk factors, and gender (operationalized as sexual orientation and gender identity) on the gut microbiome as an effector of CVD risk (Fig. 3). As stated above, there are notable sex differences in gut microbiota across a lifespan, and these differences may serve, in part, as the substrate for sex differences in CVD risk across a lifespan. The distribution of gut microbiota varies according to age (childhood, puberty, pregnancy, menopause, and old age) and sex. Also, as already established, this gut microbiota can contribute and is linked to CVD. It is critical to understand which gut microbiota and/or microbial derived metabolites may be linked to CVD in the sexes. To that end, Garcia-Fernandez *et al*. [317], analyzed gut microbiota data from the CORDIOPREV study, a clinical trial which involved 837 men and 165 women with CVD compared to their reference group of 375 individuals (270 men, 105 women) without CVD. They clearly demonstrated a sex-specific difference in beta diversity. Additional analysis showed there were sex-specific alterations in the gut microbiota linked to CVD. Women who have CVD show increased *UBA1819* (*Ruminococcaceae*), *Bilophila*, *Phascolarctobacterium*, and *Ruminococcaceae incertae sedis* while men with CVD had a higher abundance of *Subdoligranulum*, and *Barnesiellaceae*. The authors concluded that the dysbiosis of the gut microbiota associated with coronary heart disease (CHD) seems to be partially sex-specific, which may influence the sexual dimorphism in its incidence particularly since the bacteria identified to be higher in CVD patients are linked to inflammation, intestinal barrier dysfunction, and CVD directly [317, 318].

The dysbiotic gut microbiome is associated with increased blood pressure and risk of hypertension [319]. Virwani *et al*. [320], specifically examined sex differences, gut microbiota and hypertension. Interestingly they reported that significant differences in beta-diversity and gut microbiota composition in hypertensive versus normotensive groups were only observed in women and not in men. Specifically, *Ruminococcus gnavus*, *Clostridium bolteae*, and *Bacteroides ovatus* were significantly more abundant in hypertensive women, whereas *Dorea formicigenerans* was more abundant in normotensive women. Furthermore, total plasma short-chain fatty acids and propionic acid were independent predictors of systolic and diastolic blood pressure in women but not men. *Ruminococcus gnavus* and *Clostridium bolteae* have been reported to induce inflammation and are pathogenic in humans. Gut microbial-derived metabolites are likely critical to affect the way gut microbiota influences systemic disease states. As noted above, butyrate may exacerbate hypertension, as propionate has also been demonstrated in this study [160]. However, the mechanisms by which this occurs are not elucidated, but need to be to fully understand the interactions of these SCFA and hypertension outcomes in women.

In addition to sex differences in gut microbiota and CVD, there are also sex differences in many of the risk factors associated with CVD of which most have associations with the gut microbiota including diabetes, hypertension and dyslipidemia, and obesity (see review by Ahmed and Spence [321]), which may be further exacerbated by race and ethnicity [322]. In addition, sex-specific CVD risk factors related to maternal health during pregnancy may also influence and be influenced by the gut microbiome. In 2023, Colonetti *et al*. [323], conducted a meta-analysis which included 6 studies, with 479 pregnant women. They reported a significantly lower gut microbiota alpha diversity in pregnant women with pre-eclampsia in comparison with healthy controls, while no significant differences were found in the relative abundance of Bacteroidota, Bacillota, Actinomycetota, and Pseudomonadota, despite significant differences being reported in the individual studies [323]. However, this could be due to a number of factors, most significantly the analytical techniques used to identify lower levels of taxonomic resolution that vary greatly between gut microbiota studies. A rodent study by Jama *et al*. [324], examined female C57BL/6J dams fed nutrient-matched high- or low-fiber diets during pregnancy and lactation, to understand how maternal fiber influences the gut microbiota. In addition, to evaluate long-term effects and predisposition to CVD, the authors exposed 6-week-old male offspring to saline or angiotensin II for 4 weeks to induce hypertension and organ damage. Results showed that male offspring from low-fiber-fed dams had significantly larger hearts relative to body weight, and echocardiography studies in the offspring demonstrated low-fiber offspring had increased LV posterior wall thickness, confirming hypertrophy, and reduced ejection fraction, showing reduced LV contraction [324]. Regarding the gut microbiota, offspring born to dams who received a low-fiber diet showed distinct gut microbial colonization that persisted into adulthood, with higher levels of several taxa, including *Akkermansia* species. Furthermore, the authors reported that they identified 174 microbial enzymatic pathway signatures enriched in low-fiber offspring with 154 of the identified enzyme signatures in low-fiber belonged to *Akkermansia muciniphila*. *Akkermansia muciniphila*-upregulated genes encoded for mucolytic enzymes that degrade the intestinal mucus, putting the colon at risk for inflammation [324]. In contrast, high-fiber offspring had only 5 grouped enzyme signatures, which belonged to *Bacteroides ovatus*, *Escherichia coli*, and *Lactobacillus murinus*; the latter of which has been known to reduce inflammatory pathways and blood pressure. The gut microbiota of women with hypertensive disorders of pregnancy is different from that of women with normotensive pregnancy [325]. Pregnant women with hypertensive disorders of pregnancy had a higher abundance of *Rothia*, *Actinomyces*, and *Enterococcus* and a lower abundance of *Coprococcus* than pregnant women with normotension [325]. Indeed, results from Mendelian randomization support a causal relationship between gut microbiota and hypertensive disorders of pregnancy [326]. Wu *et al*. [326] found causal associations *of LachnospiraceaeUCG010*, *Olsenella*, *RuminococcaceaeUCG009*, *Ruminococcus2*, *Anaerotruncus*, *Bifidobacterium*, and *Intestinibacter* with gestational hypertension, of *Eubacterium* (*ruminantium group*), *Eubacterium* (*ventriosum group*), *Methanobrevibacter*, *RuminococcaceaeUCG002*, and *Tyzzerella3* with preeclampsia, and of *Dorea* and *RuminococcaceaeUCG010* with eclampsia, respectively. These findings are supported by experimental studies whereby fecal microbiota transplantation from preeclamptic women into preeclamptic rats significantly exacerbated the phenotype whereas the gut microbiota of healthy pregnant women had significant protective effects [327]. *Akkermansia muciniphila*, propionate, or butyrate significantly alleviated the symptoms of preeclamptic rats whereas *Akkermansia*, *Oscillibacter*, and SCFAs could be used to accurately diagnose preeclampsia [327]. Taken together, recent findings support that gut dysbiosis is important in the etiology of preeclampsia, a significant sex-specific risk factor for CVD in women.

To date there are very few studies examining gut microbiota and gender (operationalized as sexual orientation and gender identity) hence research in this area is greatly needed. Rosendale *et al*. [328], recently published a cross-sectional study of 12,180 adults using 2007–2016 National Health and Nutrition Examination Survey data, Black, Hispanic, and White sexual minority female individuals with the primary outcome of overall cardiovascular health score. Results showed that Black, Hispanic, and White sexual minority female adults had lower overall cardiovascular health scores compared with their heterosexual counterparts. Furthermore, there were no differences in overall cardiovascular health scores for sexual minority male individuals of any race or ethnicity compared with White heterosexual male individuals [328]. It is important to mention that there are even fewer studies on GAHT and gut microbiota [329], and none to our knowledge which include CVD which is an area of research importance.

## 5. Future Directions

The mantra “exercise is medicine” is often touted as a solution to restore cardiovascular health and prevent disease. Indeed, as discussed above, exercise has a powerful effect on improving gut health, attenuating vascular aging, improving large artery compliance and systemic vascular endothelial function through its antioxidant effects, and preserving nitric oxide bioavailability - all reducing the risk for CVD. However, exercise (like medicine) is not accessible to all and exercise is not medicine for all. Black adults, Hispanic adults, and women in general are not meeting physical activity recommendations. Unique social barriers such as neighborhood dynamics (safety and cohesion) may contribute to disparities in physical activity engagement across different races and ethnicities [330, 331]. There is also considerable heterogeneity in the response to exercise across race and sex [332]. For example, while women may have a blunted cardiovascular physiological response to exercise training compared to men [333], women derive greater protection against CVD mortality from that same amount of exercise [334]. Indeed, the female athlete’s heart has a lower risk of experiencing exercise-induced coronary calcification, LV fibrosis, atrial fibrillation, lethal ventricular arrhythmias and sudden cardiac death. There is also racial variation in the cardiovascular response to acute exercise and exercise training [335, 336]. Some of the differences in cardiovascular responses to exercise may be related to the physiological impact of various psychosocial factors [337, 338]. For example, racial discrimination is associated with oxidative stress and endothelial damage [339, 340]. Future research is needed to explore racial variation and sex differences in the gut microbiome’s response to exercise. Can targeting the gut with diet (e.g., prebiotics), probiotics and/or exercise confer cardiovascular resilience? Additional research is also needed to examine the effect of the gut microbiome on cardiovascular responses to exercise training. Does underlying dysbiosis mediate or moderate heterogeneity in physiological adaptations to exercise training? Additional research will also be needed to understand the importance of intersectionality on the gut microbiome, considering race, ethnicity, sex and gender.

## 6. Conclusions

Studies continue to support that gut dysbiosis is a CVD risk factor, with numerous microbes impacting unique aspects of cardiovascular structure and function. The gut microbiome is shaped by biological sex, gender, race and ethnicity, potentially contributing to cardiovascular health disparities and sex differences in CVD. Psychosocial factors related to systemic racism, sexism, and discrimination impact the microbiome via effects on diet and food access. These same factors may also activate physiological stress systems, contributing to inflammation, oxidative stress, subclinical changes in vascular structure and function (i.e., EDD and arterial stiffening) and ultimately CVD.

To conclude, sociology impacts physiology and contributes to pathophysiology. Oppressive social factors experienced by minorities and women may shape the gut, in turn contributing to cardiovascular health disparities. Exercise remains a critical lifestyle and biobehavioral factor to promote gut resilience and foster cardioprotection.
